# CD4^**+**^CD25^**+**^ Regulatory Cells Contribute to the Regulation of Colonic Th2 Granulomatous Pathology Caused by Schistosome Infection

**DOI:** 10.1371/journal.pntd.0001269

**Published:** 2011-08-09

**Authors:** Joseph D. Turner, Gavin R. Jenkins, Karen G. Hogg, Sarah A. Aynsley, Ross A. Paveley, Peter C. Cook, Mark C. Coles, Adrian P. Mountford

**Affiliations:** Centre for Immunology and Infection, Department of Biology, The University of York, York, United Kingdom; Uniformed Services University, United States of America

## Abstract

Eggs of the helminth *Schistosoma mansoni* accumulate in the colon following infection and generate Th2-biassed inflammatory granulomas which become down- modulated in size as the infection proceeds to chronicity. However, although CD4^+^CD25^+^FoxP3^+^regulatory T cells (T_regs_) are known to suppress Th1-mediated colitis, it is not clear whether they control Th2 –associated pathologies of the large intestine which characterise several helminth infections. Here we used a novel 3D-multiphoton confocal microscopy approach to visualise and quantify changes in the size and composition of colonic granulomas at the acute and chronic phases of *S. mansoni* infection. We observed decreased granuloma size, as well as reductions in the abundance of DsRed^+^ T cells and collagen deposition at 14 weeks (chronic) compared to 8 weeks (acute) post-infection. Th2 cytokine production (*i*.*e.* IL-4, IL-5) in the colonic tissue and draining mesenteric lymph node (mLN) decreased during the chronic phase of infection, whilst levels of TGF-β1 increased, co-incident with reduced mLN proliferative responses, granuloma size and fibrosis. The proportion of CD4^+^CD25^+^FoxP3^+^T_regs_: CD4^+^ cells in the mLN increased during chronic disease, while within colonic granulomas there was an approximate 4-fold increase. The proportion of CD4^+^CD25^+^FoxP3^+^T_regs_ in the mLN that were CD103^+^ and CCR5^+^ also increased indicating an enhanced potential to home to intestinal sites. CD4^+^CD25^+^ cells suppressed antigen-specific Th2 mLN cell proliferation *in vitro*, while their removal during chronic disease resulted in significantly larger granulomas, partial reversal of Th2 hypo-responsiveness and an increase in the number of eosinophils in colonic granulomas. Finally, transfer of schistosome infection-expanded CD4^+^CD25^+^T_regs_ down-modulated the development of colonic granulomas, including collagen deposition. Therefore, CD4^+^CD25^+^FoxP3^+^T_regs_ appear to control Th2 colonic granulomas during chronic infection, and are likely to play a role in containing pathology during intestinal schistosomiasis.

## Author Summary

Schistosomiasis is an important parasitic helminth disease afflicting more than 200 million people worldwide. Infections are typically chronic and in the case of *Schistosoma mansoni* and *S. japonicum* the majority give rise to an intestinal form of disease caused by the deposition of parasite eggs in the colon and terminal ileum. The eggs cause Th2-associated inflammatory immune granulomas to form, which as the disease develops, are down-regulated by cells of the immune system. However, the mechanisms which underpin the down-regulation of granulomas in the large intestine are not known. In order to investigate the phenomenon of Th2-associated colonic inflammation, we utilized a murine model of infection with *S. mansoni* and compared immune responses at the acute and chronic phases of infection. We show that a type of regulatory T helper lymphocyte (CD4^+^CD25^+^FoxP3^+^T_reg_) contributes to regulation of colonic inflammation. These cells modulate anti-egg Th2 responses within the mesenteric lymph nodes and granulomatous pro-fibrotic Th2 responses within the colon. Our study highlights the importance of CD4^+^CD25^+^FoxP3^+^T_regs_ as a source of regulatory pressure on granuloma formation in the colon and by implication humans with chronic intestinal schistosomiasis.

## Introduction

Schistosomiasis is an important parasitic helminth disease afflicting more than 200 million people, causing approximately 280 thousand deaths annually, with a further estimated 700 million at risk of infection [1], [2]. In the case of *Schistosoma mansoni,* infections are typically chronic (>10 years) and the majority (>90%) give rise to an intestinal form of disease [3] caused by the deposition of parasite eggs in the intestinal mesenteries (mainly of the colon and terminal ileum) and the subsequent development of Th2-associated granulomatous infiltrates rich in macrophages and eosinophils [4]. Such infections lead to diarrhoea, pseudopolyposis, microulceration, bleeding and fibrosis [5]. Recent re-appraisal of Disability-Associated Life Years (DALYs) attributable to schistosomiasis, where more subtle disease manifestations such as intestinal schistosomiasis have been included, raises the disease burden caused by this infection as much as 40-fold, putting schistosomiasis on a par with malaria as a global public health problem [6]. Variation in granuloma size in the colon between patients is positively associated with peripheral blood mononuclear cell (PBMC) reactivity to soluble egg antigens (SEA) [7]. Thus, changes in lymphocyte responsiveness appear to be related to the size of granulomas in the intestine and by implication, the severity of pathologies in patients with intestinal disease.

In order to investigate the phenomenon of Th2-associated colonic inflammation and possible mechanisms underlying its regulation, we utilized a murine model of infection with *S. mansoni* which provides a well accepted permissive experimental host. In the murine model, myeloid antigen presenting cells, including dendritic cells [8], [9], and basophils [10], are primed to induce potent anti-egg Th2 CD4^+^ lymphocyte responses. Th2 activation appears necessary to protect the host from lethal hepatic and intestinal damage during acute infection [11] and to keep Th1 inflammatory immunopathology in check [12]. However, survival to the chronic stage of infection, representative of human disease, is dependent on modulation of the Th2 granulomatous response in order to subvert IL-4/IL-13-driven morbidity [13]. ‘Naturally occurring’ (n)T_regs_ bearing the IL-2 receptor α chain molecule (CD25) and expressing the transcription factor forkhead box P3 (FoxP3) have been demonstrated to play a role in the regulation of Th2 anti-egg hepatic inflammation in an IL-10-independent manner [14], [15], although their role in regulating intestinal inflammation induced by egg deposition has not been determined.

Our data presented herein support a role for CD4^+^CD25^+^FoxP3^+^T_regs_ in regulating colonic inflammation by modulating both anti-egg Th2 responses within the mesenteric lymph nodes (mLN) and granulomatous, pro-fibrotic Th2 responses within the colon. Thus, our study implicates CD4^+^CD25^+^FoxP3^+^T_regs_ as a source of regulatory pressure during chronic intestinal schistosomiasis and in the wider context, as suppressors of Th2-driven pathology in the colon.

## Materials and Methods

### Ethics statement

All experiments were carried out in accordance with UK Animal's Scientific Procedures Act 1986 and with the approval of The University of York Ethics Committee.

### Experimental infection and parasitological readout

C57BL/6 (B.6) and hCD2-VaDsRed-B.6 mice were maintained within the University of York under specific pathogen-free conditions. hCD2-DsRed-B6 mice, were a gift of D. Kioussis and A. Patel (National Institute for Medical Research, London) and express fluorescent DSRed T cells (>90% CD3+) to facilitate in situ detection of T cells by multiphoton microscopy (see below). Eight to ten-week female mice were infected percutaneously via the abdomen with 25 *S. mansoni* cercariae, and infections allowed to mature for either 8 or 14 weeks representing the acute and chronic phases of infection respectively. Adoptive transfer recipients were infected with 100 cercariae. Egg burdens in the 5 cm of colon proximal to the cecum were enumerated following digestion in 4% KOH. Eggs in faecal material were enumerated following dispersion in PBS, filtration through 100 µm pore mesh, and concentration. Colonic granulomas were isolated as previously described [16]. Volumes were calculated by measuring the longest and widest points and extrapolating volume using standard formulae for sphere or cylinder, depending on individual granuloma shape.

### Histology and confocal microscopy

*C*olonic tissue were fixed in 4% formaldehyde and embedded in wax. Transverse cross-sections (5 µm) were stained with H&E, or haemotoxylin and Van Geison (Department of Veterinary Pathology, University of Liverpool). Digital photomicrographs were analysed using AxioVision software (Zeiss).

For multiphoton imaging, proximal colon segments were mounted within 10 mm depression slides, and granulomas imaged from the serosal surface to egg mid-point using a 510 NLO laser-scanning microscope (LSM, Zeiss) with multi-photon laser (Coherent) tuned to 872 nm. 3D projections of ‘half-granulomas’ were rendered from z stacks using Volocity 4 software (Improvision). Quantification of Ds-Red^+^ lymphocytes, granuloma and collagen volumes were performed using “ROI” and “RGB” measurement tools within Volocity.

For immunofluorescent staining, frozen tissues were cryosectioned at 8 µm intervals, fixed with 10% methanol, permeabilised with 0.5% saponin (Sigma), and blocked with 5% rabbit serum / 1% FCS. Sections were labelled with anti-CD4 AF488 and anti-FoxP3 AF647 (both eBioscience) and fluorescence captured using the 510 NLO LSM. Settings for acute and chronic fluorescence images are matched both with respect to laser scanning settings at the time of image capture and post-image digital enhancement. Baseline laser scanning settings were undertaken on isotype controls and resultant negative control images contain undetectable fluorescent signal.

### Anti-CD25 mAb treatment

Three doses of anti-CD25 mAb (50 µg; clone PC61, a gift from F. Powrie, University of Oxford), or purified rat IgG2a, were delivered intraperitoneally to infected mice at 9, 11, and 13 weeks.

### T_reg_ cell purification, adoptive transfer and *in vitro* culture

T_regs_ from the mLN were purified by depletion of non-CD4^+^ cells followed by isolation of CD25^+^ cells using antibodies conjugated to magnetic beads (Miltenyi Biotec). For adoptive transfer, 2.5×10^6^ CD4^+^CD25^+^T_regs_ (>90% purity) were injected via the lateral tail vein. Total mLN cells (2×10^6^/ml), sorted CD4^+^CD25^−^ effector cells (1×10^6^/ml), and CD4^+^CD25^+^T_regs_ (0.5×10^6^/ml) from infected mice cultured in complete RPMI-1640 medium (containing 10% FCS, 50 µg/ml penicillin/streptomycin), in combination with naïve mLN CD4^−^CD25^−^ cells (0.1×10^6^/ml) as a source of APC. Cells were stimulated with plate-bound anti-CD3 mAb (1 µg; Becton Dickinson), or SEA (50 µg/ml) [16]. Cells were cultured for 72 h and supernatants retained for cytokine analysis. Proliferation was measured from 72 to 96 h by ^3^H-thymidine incorporation and scintillation counting.

### Cytokine and collagen quantifications

ELISAs were used to quantify IL-4, IL-5 and IFNγ [17], while IL-10 and IL-13 were measured by Cytoset (Invitrogen) or DuoSet (R&D Systems) kits respectively. A TGFβ-sensitive, mink lung epithelial cell bio-assay (MLEC transfected with firefly luciferase; gift from Daniel Rifkin, NY Medical Center) was used to determine levels of bio-active TGFβ1 [18]. As the bio-assay was not compatible with tissue extracts, a TGFβ1 ELISA (R&D Systems) was employed. In order to determine cytokine levels in the colon, frozen tissues were first homogenised in proprietary tissue extraction buffer containing detergent and protease inhibitors (Thermo Scientific) and then incubated/rotated overnight at 4°C and the soluble fractions isolated by centrifugation prior analysis by ELISA. Salt-soluble collagen was quantified using colorimetric assay (Sircol, Biocolor).

### Quantitative Real Time PCR

Total colonic mRNA was used to generate cDNA using Superscript III DNA polymerase (Invitrogen) and *foxp3* transcript analysed by qRT-PCR (ABI PRISM 7000; Applied Biosystems) using Taqman probes (Sigma-Aldrich). The relative expression of *foxp3* was normalised to values obtained for *cd3*. Primer pairs and probes were; *foxp3* 5′-GCAGTGTGGACCGTAGATGA, 5′-CACAGCCTCAGTCTCATGGT, Probe 5′-ACAAGTGCTCCAATCCCTGCCCTT and *cd3* 5′-GAGCACCCTGCTACTCCTTG, 5′- ATGTCCCAGCACTGGCTACT, Probe 5′- TGCTCTTCAGCCTCCTGGTGAACAC.

### Flow Cytometry

Cells were blocked with anti-CD16/CD32 (eBioscience) at 0.5 µg / 1×10^6^ cells, then labelled with anti-CD4-Pacific Blue, anti-CD25-APC (PC-61), anti-CD103-PE (all eBioscience), anti-CD25-FITC (7D4), anti-CTLA-4-FITC, or anti-CCR5-biotin (BD Bioscience) for 30 minutes. Biotinylated antibodies were sequentially detected with streptavidin-PE-Cy7 (eBioscience). For intracellular staining of FoxP3, cells were fixed in 1% formalin, re-suspended in permeablisation buffer (Becton Dickinson) prior to labelling with anti-FoxP3-PE or -AF647 (eBioscience). Cells were analysed using a Cyan flow cytometer with Summit software (Beckman Coulter).

### Statistical analyses

Significant differences between two experimental groups were determined by unpaired Student's T test, and between three or more groups by 1-way ANOVA with Tukey post-hoc tests using Prism software (GraphPad). Because colonic egg counts were skewed, analysis was undertaken after Log10 transformation. All data are representative of a minimum of two independent experiments. Significance is indicated ***P<0.001, **P<0.01, *P<0.05. Significance values are shown on the figures with line connectors between the appropriate groups. Where statistical significance was not achieved (P>0.05), figures are intentionally left blank.

## Results

### Colonic granuloma size and anti-egg Th2 responses decline with chronicity of infection

**Figure 1:**
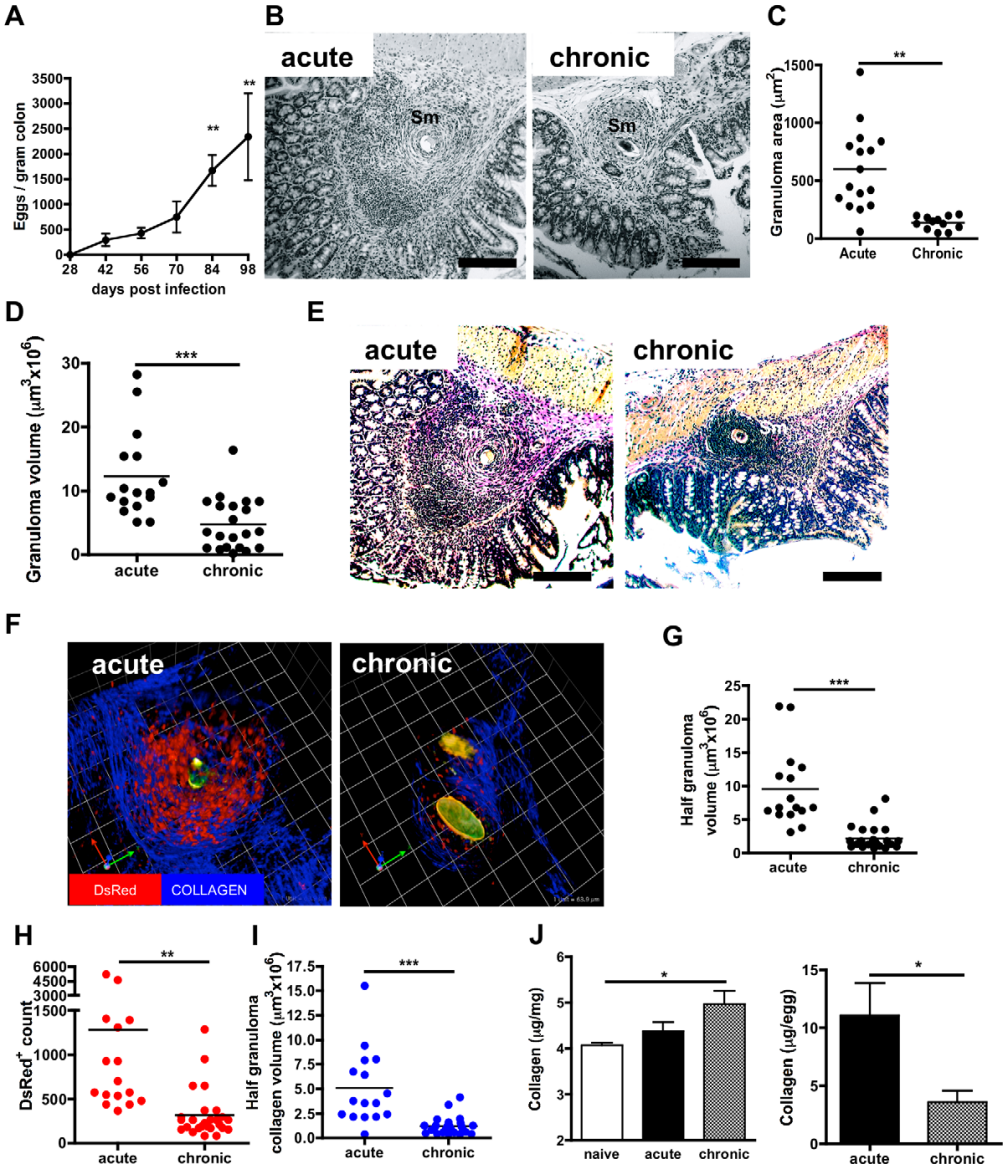
Colonic granuloma size and fibrosis is reduced in the chronic phase of schistosome infection. **A).** Accumulation of eggs /gram of colon tissue (n = 4 mice/time point); mean eggs (± SEM). **B**) Representative photomicrographs of the colon at the acute (8 wks) or chronic (14 wks) stage of infection stained with H&E. Scale bars are 200 µm: egg denoted ‘Sm’. **C**) Granuloma area at the acute or chronic stage; bars are mean / animal (n = 4) from three separate histological sections. **D**) Granuloma volumes calculated from isolated granulomas at the acute or chronic stage calculated from three separate granulomas per individual animal. **E**) Cross sections of colon at the acute (8 wks) or chronic (14 wks) stage of infection stained with haematoxylin / Van Geison (collagen fibres  =  pink). All scale bars are 200 µm. Parasite egg is denoted ‘Sm’. **F**) 3D images of multiphoton confocal stacks of colonic tissue from infected hCD2-VaDsRed-B.6 mice sampled *in situ* during acute or chronic infection. DsRed fluorescent cells are >90% CD3^+^; blue fluorescence is second harmonic generation of type 1 collagen; green/yellow auto-fluorescence are schistosome eggs. Grid squares are 63.9 µm^2^. Quantification of multiphoton confocal stacks from hCD2-VaDsRed-B.6 mice: **G**) granuloma half-volumes, **H**) DsRed^+^ cell counts and **I**) collagen half-volumes. Data calculated from four separate granulomas from individual animals (n = 4). **J**) Salt-soluble collagen from colonic tissues of naïve, acute, and chronic mice (n = 4). Data is mean (±SEM) collagen conc^n^–/mg of tissue (left), or adjusted for numbers of eggs/mg (right).

Egg deposition and anti-egg granulomatous responses in proximal colons were examined over a time-course of infection in B.6 mice. The numbers of eggs increased during infection from 292±121.8 (day 42) to 2339±863.5 (day 98) (Fig. 1A). Mean areas of isolated colonic granulomas declined from the acute to chronic time point visualised by H&E staining (Fig. 1B & C), supporting previous observations [16], [19]. Estimates of volumes of granulomas isolated from enzymatically digested colons (Fig. 1D) corroborated histological observation and showed a significant decrease in size between the acute and chronic stage of infection. In addition, the decrease in granuloma size at the chronic stage was accompanied by a decrease in collagen, indicative of fibrosis, as shown by Van-Geison stained sections (Fig. 1E).

Multiphoton imaging of proximal colon derived from infected hCD2-VaDsRed-B.6 mice revealed further quantitative information on temporal granuloma modulation in situ (Fig. 1F, Videos S1 & S2) and analysis of 3D images showed granuloma volumes were significantly decreased at the chronic stage (Fig. 1G). Furthermore, numbers of granuloma-associated DsRed^+^ lymphocytes (Fig 1H; >90% CD3^+^T lymphocytes, data not shown), and granuloma-associated type-1 collagen deposition, revealed as second harmonic imaging (blue), was significantly also reduced (Fig. 1I). Although a significant increase in the recently synthesised (salt-soluble) collagen pool within the colon was apparent by the chronic phase, modulation of the egg-driven fibrotic response was demonstrable when adjusted for the increased numbers of eggs (as a surrogate for numbers of granulomas) in chronic infected colons (Fig. 1J).

**Figure 2:**
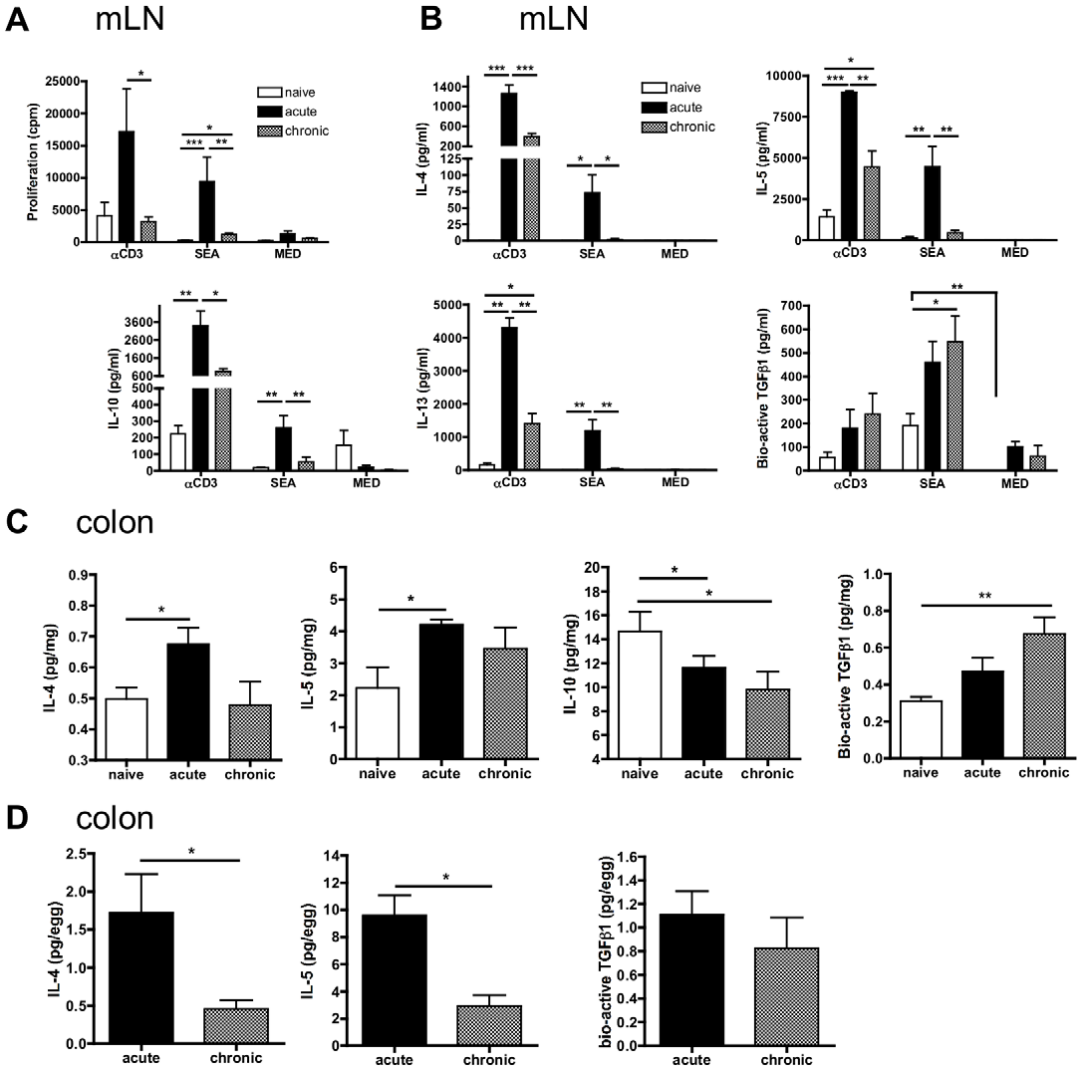
Egg antigen-specific Th2, but not TGF-β1 responses become down-modulated within the mLN and colon during chronic infection. **A)** Proliferative responses and **B)** cytokine release (pg/ml) by mLN cells from naïve, acute, or chronic mice (n = 4/group) to anti-CD3 mAb, or SEA. **C)** Cytokine levels within colonic tissues (pg/mg tissue) and **D)** adjusted for numbers of eggs / mg of tissue. Data are mean proliferative response / cytokine concentration ±SEM.

At the acute stage, anti-CD3 mAb and SEA-specific proliferation of mLN cells were significantly elevated and biased towards secreting Th2-type cytokines (Fig. 2A). However, by the chronic stage, SEA-induced cell proliferation and production of IL-4, IL-5 and IL-13 were significantly reduced (Fig. 2B). While the secretion of IL-10 in response to SEA was significantly lower during chronic compared to acute infection the production of bio-active TGFβ1 to SEA at the chronic stage was significantly elevated compared to naïve state (Fig. 2B).

It was not possible to obtain sufficient numbers of viable lymphocytes via enzymatic digestion of granulomatous colons due to the fibrotic nature of these intestinal granulomas but levels of IL-4 and IL-5 in whole colonic extracts were elevated at the acute phase (Fig. 2C), suggesting that tissue inflammatory responses in infected colons mirrored the Th2 response in the mLN. Surprisingly, levels of IL-10 significantly decreased in infected colonic tissue (Fig. 2C) but levels of bio-active colonic TGFβ1 were elevated during chronic disease. When adjusted for numbers of deposited eggs, production of colonic IL-4 and IL-5 was significantly diminished at the chronic phase (Fig. 2D). Thus, whilst local cytokine responses to egg deposition has both shared and distinct facets to those of the mLN, measurements indicate that colonic Th2 responses establish during the onset of egg deposition and subsequently diminish as chronicity proceeds.

### Increased numbers of CD4^+^FoxP3^+^T_reg_ cells occur in the mLN and colonic granulomas as chronicity proceeds

**Figure 3:**
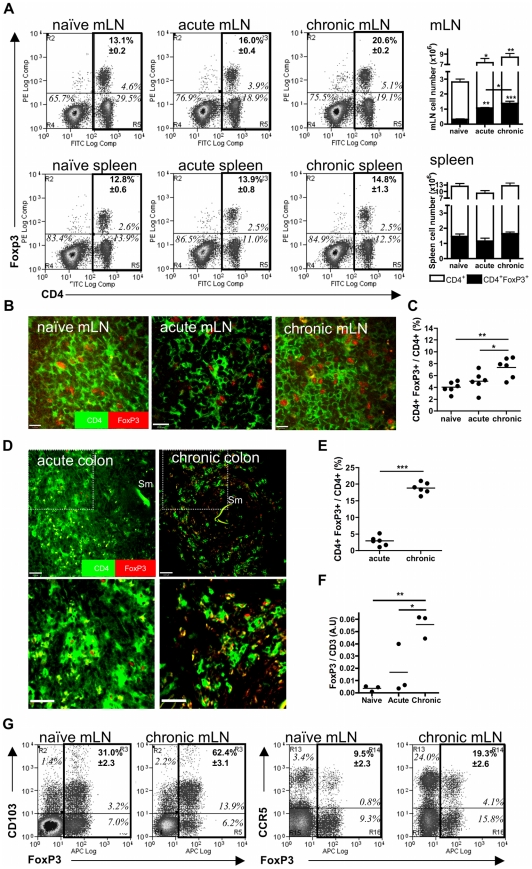
Frequencies of CD4^**+**^FoxP3^**+**^ T_regs_ increase at the chronic phase in both the mLN and colonic granulomas. **A**) Representative flow cytograms showing the frequencies of labelled CD4^+^ and FoxP3^+^ cells in suspensions of the mLN and spleen. Values in italics are quadrant percentages. Values in bold, upper right-hand quadrant, are mean (±SEM) CD4^+^FoxP3^+^ cells as a % of total CD4^+^ cells (relevant quadrants outlined in bold). Histograms show mean (±SEM) total CD4^+^ (open) and CD4^+^FoxP3^+^ (closed), mLN or spleen cell or numbers (n = 3 mice). Significant differences compared with naïve cell numbers are indicated. Bar indicates significant difference between CD4^+^FoxP3^+^ mLN numbers at the chronic compared with acute stage. **B**) Representative images of CD4^+ (^green) and FoxP3^+^ (red) cells in labelled cryosections of mLN and, **C**) proportion of CD4^+^FoxP3^+^ / CD4^+^ cells/ field of view (2 fields of view / animal, n = 3) Scale  = 14 µm. **D**) Representative images of CD4^+^ and FoxP3^+^ cells in colonic granulomas, with high power insert also shown. **E**) Frequencies of labelled CD4^+^FoxP3^+^ / CD4^+^ colonic granuloma cells / field of view (2 fields of view / animal, n = 3). **F**) qRT-PCR analysis of FoxP3 mRNA in colonic tissue plotted as arbitrary units of FoxP3 normalised to CD3 transcript in colonic tissue. Bars are mean FoxP3 A.U. transcript per group. **G).** Flow plots of FoxP3 and CD103, and FoxP3 and CCR5 expression, gated on CD4^+^ mLN cells of naïve or chronic mice (n = 4). Values in italics are quadrant percentages. Values in bold, upper right-hand quadrant, are mean (± SEM) CD4^+^FoxP3^+^CD103^+^ cells, or CD4^+^FoxP3^+^CCR5^+^ cells as a % of total CD4^+^FoxP3^+^ cells (relevant quadrants outlined in bold).

The proportion of CD4^+^ FoxP3^+^T_regs_ in the mLN as a proportion of total CD4^+^ cells, as determined by flow cytometry of cell suspensions, increased from 13.1±0.2% in naïve mice to 16.0±0.4% during acute infection (P<0.001), and increased further to 20.6±0.2% during chronic infection (P<0.001; Fig. 3A). Absolute numbers of both mLN CD4^+^ effector and CD4^+^FoxP3^+^T_reg_ cells increased during acute disease from naïve levels, and remained significantly elevated during chronic infection (Fig. 3A). The increase was confirmed by enumeration of FoxP3 T_regs_ in stained sections of mLN from naïve mice and those with acute and chronic infection (Fig. 3B & C). In contrast, absolute numbers or proportions of CD4^+^FoxP3^+^T_regs_ in the spleens did not expand (12.8±0.6% *cf*. 13.9±0.8% *cf.* 14.8±1.3%, Fig. 3A).

A pronounced increase in the proportion of CD4^+^FoxP3^+^T_regs_ within colonic granulomas at the chronic phase of infection, from 2.9±0.6% to 18.8±0.7% was revealed by enumeration of double positive versus single positive cells in anti-CD4 / anti-FoxP3 immunostained cryosections of colonic tissue (Fig. 3D & E). This profound (>10 fold) proportional elevation in FoxP3^+^ cells compared with total number of T lymphocytes in gut tissue was corroborated by qRT-PCR of FoxP3 transcript normalised to CD3 transcript (Fig. 3F). Thus, during enteric *S. mansoni* infection, relative and absolute expansion in the numbers of CD4^+^FoxP3^+^ cells occurs preferentially within gut-associated lymphoid tissue (GALT). Furthermore, relative increases of CD4^+^FoxP3^+^ cells within colonic granulomas are apparent during chronic disease.

CD103, the αE molecule of the αEβ7 mucosal integrin involved in homing of T cells to intestinal sites [20], increased on mLN CD4^+^FoxP3^+^T_regs_ at the chronic stage compared to naïve mice (31.0±2.3% *cf.* 62.4±3.1%, P<0.001, Fig. 3G). CCR5 is also involved in T cell homing to intestinal inflammatory sites [21], and significantly, the proportion of CCR5^+^CD4^+^FoxP3^+^T_regs_ in the mLN increased markedly (9.6±2.3% *cf* 19.3±2.6%; Fig. 3G). Thus, increases in the number of mLN CD4^+^FoxP3^+^T_regs_ expressing CD103 and CCR5 suggests these cells have enhanced potential to be recruited and retained within the colonic infection site during chronic infection.

### The majority of Schistosome-expanded CD4^+^CD25^+^mLN cells are FoxP3^+^ and suppress antigen-specific CD4^+^ proliferative responses *in vitro*

**Figure 4:**
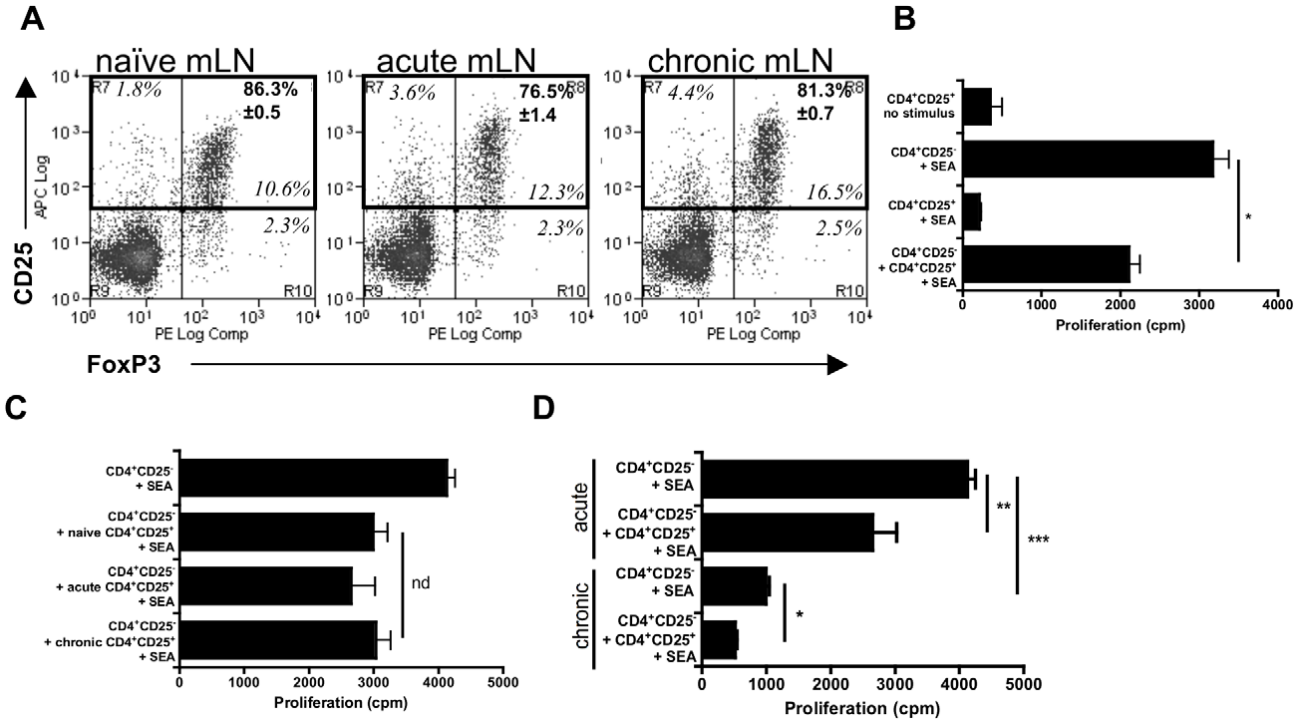
CD4^**+**^CD25^**+**^ mLN cells suppress antigen-specific CD4^**+**^ Th2 responses *in vitro.* **A**) Flow plots of mLN cell suspensions (n = 3 mice) labelled with anti-CD25 and anti-FoxP3, gated on CD4 expression. Values in italics are quadrant percentages. Values in bold, upper right-hand quadrant, are mean (± SEM) CD4^+^CD25^+^FoxP3^+^ cells as a % of total CD4^+^CD25^+^ cells (relevant quadrants outlined in bold). **B**) Antigen-specific proliferation of CD4^+^CD25^-^ effector cells from acute mice and CD4^+^CD25^+^T_regs_ from chronic mice, or co-cultured together in a 2∶1 ratio. **C**) Co-culture of CD4^+^CD25^-^ effector cells derived from acute mice cultured with CD4^+^CD25^+^T_regs_ derived from naïve, acute or chronic mice. **D**) CD4^+^CD25^-^ effector cells from mice with an acute or chronic infection, either depleted of CD4^+^CD25^+^ cells, or with CD4^+^CD25^+^ cell ‘add-back’ co-cultures from the same stage of infection at a 2∶1 ratio. All histograms are mean (± SEM) cpm ^3^H-thymidine incorporation.

More than 75% of CD4^+^CD25^+^ mLN cells co-expressed FoxP3, regardless of the stage of infection confirming that the majority of CD4^+^CD25^+^ cells can be classified as CD4^+^FoxP3^+^T_regs_ (Fig. 4A). In addition, while CD4^+^CD25^−^effector cells from mice with an acute infection exhibited a strong proliferative response in vitro to SEA, CD4^+^CD25^+^T_regs_ taken at the chronic stage of infection did not proliferate (Fig. 4B). Moreover, co-culture of these two cell populations in a 2∶1 ratio prevented optimum antigen-specific proliferation of CD4^+^CD25^−^ effector T cells (Fig. 4B). Thus, CD4^+^CD25^+^ (FoxP3^+^) cells within the mLN during chronic schistosome infection displayed a regulatory phenotype in vitro.

CD4^+^CD25^+^ T_regs_ isolated from the mLN of naïve mice, compared to mice with an acute or chronic infection, exerted similar degrees of suppression on the acute-stage anti-SEA CD4^+^ T cell proliferative response (Fig. 4C). Moreover, following depletion of CD4^+^CD25^+^ T_regs_, or after their re-addition in a 1∶2 T_reg_ / effector T cell ratio, we observed that while the T_regs_ conferred a significant degree of suppression on the anti-SEA response, chronic CD4^+^ CD25^−^ effector T cells remained hypo-responsive, or anergic, compared with their acute-stage counterparts (Fig. 4D). Taken together, these in vitro assays provide evidence that schistosome-expanded CD4^+^CD25^+^T_regs_ suppress the pre-dominant Th2 anti-SEA response. However, compared with T_regs_ from naïve mice, they are not enhanced in their ability to suppress antigen-specific CD4^+^ proliferation.

### Anti-CD25mAb treatment impairs the regulation of colonic granuloma size

Depletion of CD25^+^ cells is a common technique to experimentally induce Treg deficiency [14],[22],[23],[24],[25],[26]. Whilst not all CD25^+^ cells are FoxP3^+^ Tregs (75%–80% in our infection model) and FoxP3^+^Treg populations can be rapidly induced following CD25^+^ depletion during infection [27], a single antibody treatment with anti-CD25 clone PC61 can significantly reduce FoxP3^+^ cells by 70% [28], with reduced FoxP3^+^ cells persisting in the face of ensuing inflammation for two weeks [27]. We therefore treated 9 week schistosome infected mice with a regimen of PC61 once per two weeks for six weeks and assessed CD25^+^ lymphocyte depletion and effects on intestinal granuloma parameters one week (+14 weeks infected) following the last PC61 antibody treatment.

**Figure 5:**
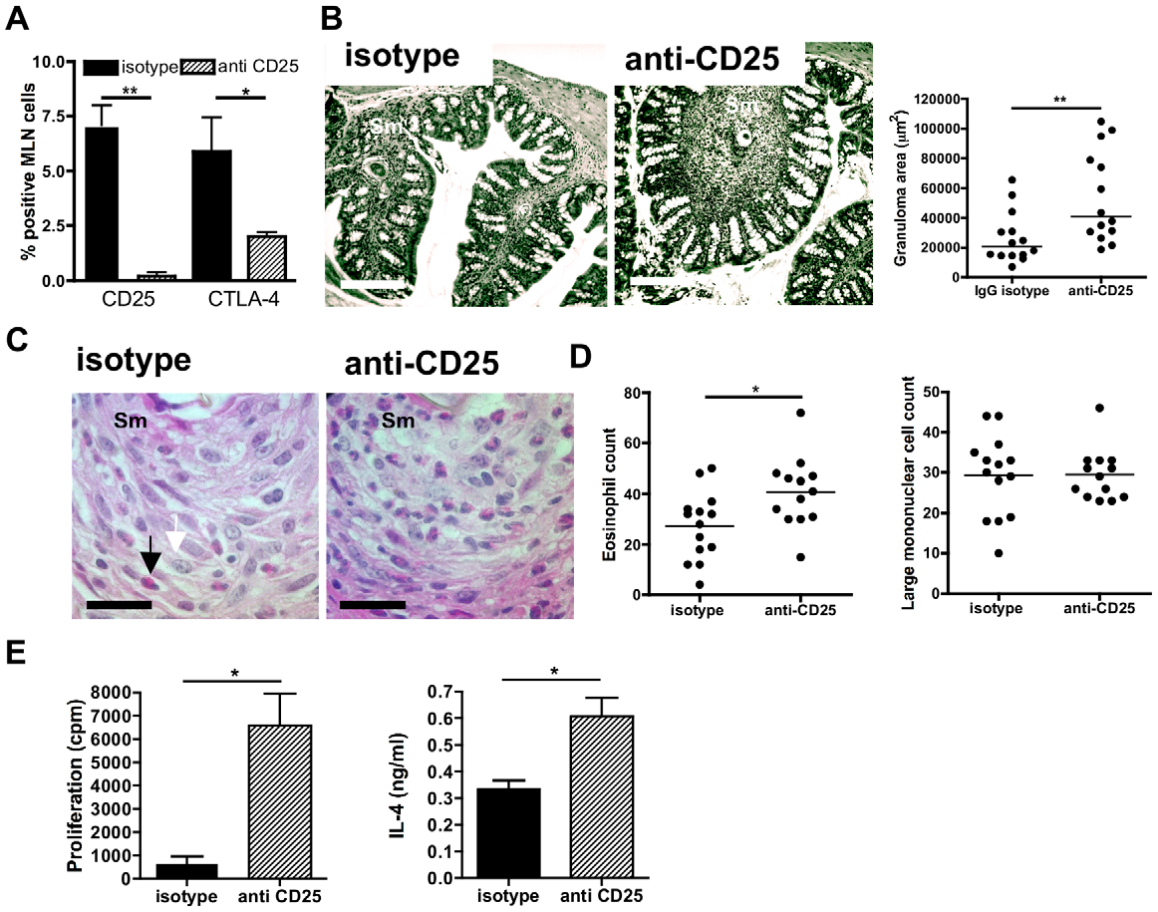
*In vivo* ablation of CD25^**+**^ cells impairs regulation of colonic granulomas and antigen-specific Th2 responses. **A**) Percentage of CD25^+^ or CTLA-4^+^ mLN lymphocytes from mice with chronic infection after treatment with anti-CD25 mAb, or isotype control. Antibodies given at 2 week intervals from wk 9 to wk 13, tissues sampled at wk 14. Data are mean % positive (± SEM). **B**) Photomicrographs of colonic tissue and quantification of granuloma areas after antibody treatment. Scale bar  = 200 µm; egg denoted ‘Sm’. Data is granuloma area (µm^2^) at wk 14 calculated from 3-4 separate histological sections per individual animal (n = 4). **C)** Colonic granulomas (x100) stained with H&E showing eosinophils (closed arrow) and large mononuclear cells (open arrow) in isotype mAb treated (left) and anti-CD25 treated (right|) infected mice. Scale bar  = 200 µm; egg denoted ‘Sm’. **D**) Numbers of eosinophils (left) and large mononuclear cells enumerated from the sections above; Bars are mean cell counts / group (n = 4) with 3–4 fields of view / mouse. **E**) Antigen-specific mLN responses in anti-CD25 mAb treated mice; data are means of cpm^ 3^H-thymidine incorporation and IL-4 secretion pg/ml (n = 3).

Anti-CD25 mAb treatment of infected mice effectively depleted CD25^+^ cells in the mLN, and reduced the proportion of CTLA-4^+^ lymphocytes (Fig. 5A). Anti-CD25-treated mice also retained significantly larger granulomas in chronic colonic tissues compared to their isotype control cohorts (P<0.01; Fig. 5B). Eosinophils adjacent to schistosome eggs were significantly more numerous in anti-CD25 treated mice, while the numbers of large mononuclear cells remained stable (Fig. 5C & D). This suggests that reductions in CD25^+^ cells, the majority of which are FoxP3^+^T_regs_, lifted suppression of eosinophil recruitment, or their retention within colonic granulomas. It was also co-incident with increased antigen-specific mLN cell proliferation and IL-4 production at the chronic phase of infection (Fig. 5E). However, in CD25^+^-depleted mice, colonic granulomas remained, on average, significantly smaller in area compared to those at the acute stage of infection (43,397±5477 µm^2^ compared with 107,128±12062 µm^2^, P<0.01). Thus, in vivo depletion of CD25^+^ lymphocytes partially, yet significantly, reverses the down-modulation of Th2 granulomatous pathology in the colon during chronic *S. mansoni* infection.

### Transfer of infection-expanded CD4^+^CD25^+^T_regs_ modulates acute colonic granuloma development

**Figure 6:**
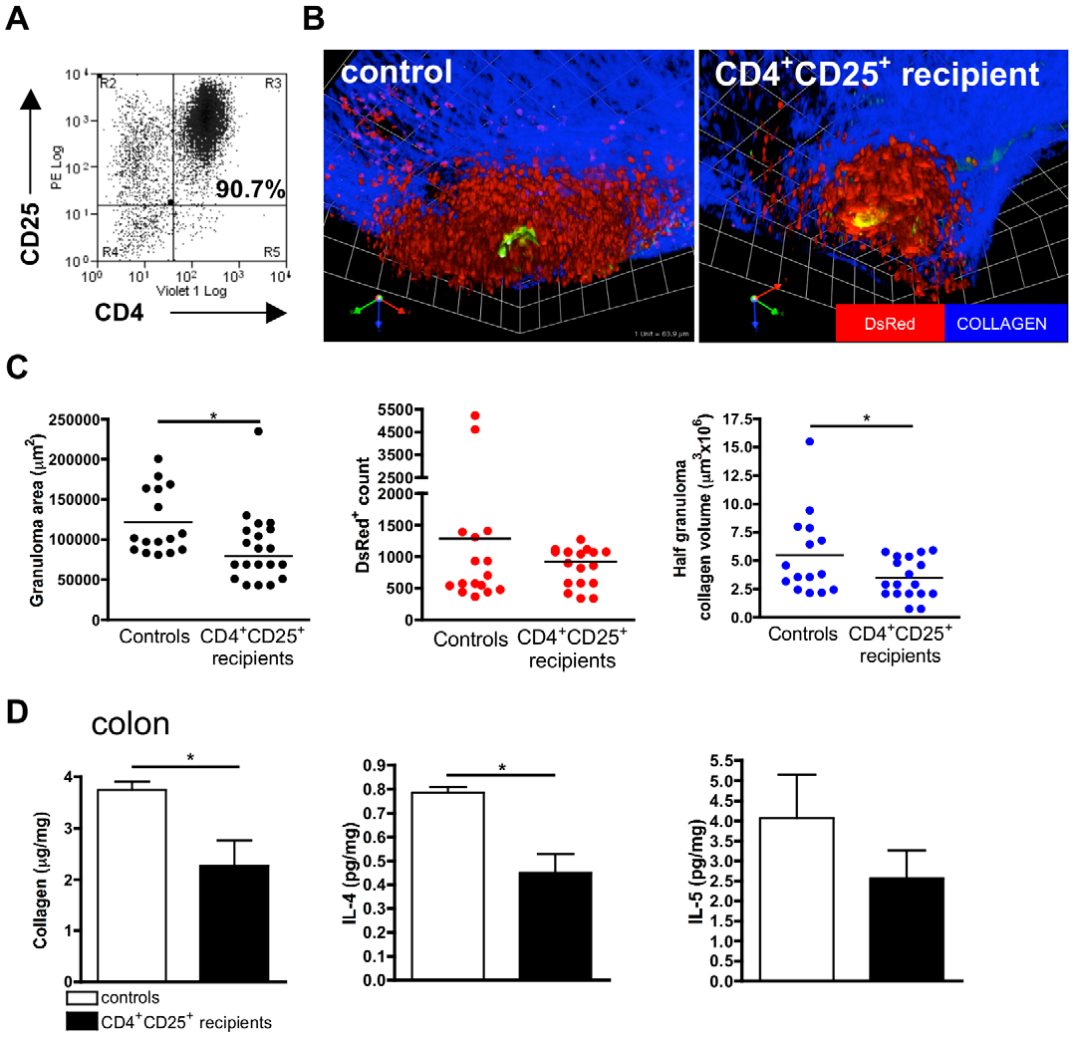
Transfer of schistosome-expanded CD4^**+**^CD25^**+**^ T_regs_ modulates the development of acute-stage granulomas. **A)** Isolated mLN CD4^+^CD25^+^T_regs_ from mice with chronic infection used for transfer. **B)** 3D images of multiphoton confocal stacks of colonic tissue viewed *in situ* at the acute stage of infection in hCD2-VaDsRed-B.6 control mice, or recipients of infection-expanded CD4^+^CD25^+^T_regs_ (2.5✕10^6^). Grid squares are 63.9 µm2. **C)** Granuloma area (left), DsRed^+^ cell counts (middle), and collagen half-volume (right) taken from confocal stacks (as above). Data are from 5–6 separate granulomas per mouse. Bars are mean / group (n = 3). **D)** Soluble collagen and cytokine in colonic extracts from infected control mice, or recipients of CD4^+^CD25^+^ cells. Bars are means / group (n = 3). Data is mean pg/ml (± SEM) per group.

Purified CD4^+^CD25^+^ mLN cells (2.5×10^6^, >90% purity; Fig. 6A) from mice at the chronic stage of infection were administered to hCD2-VaDsRed-B.6 mice co-incident with the onset of egg deposition. Four weeks later (+9 weeks post-infection), the number of eggs in tissues or excreted from CD25^+^ cell recipients were not significantly different compared with controls (Table S1). This showed that immune cell transfers did not significantly affect adult worm development, fecundity, or egg transmission. Recipients of CD4^+^CD25^+^ cells had significantly smaller granuloma area and collagen deposits but Ds-Red^+^ T cell numbers within the colonic granulomas were not significantly altered (Fig. 6B &C; Videos S3 & S4). Recipients also had decreased levels of recently synthesised collagen and IL-4 in colonic extracts (Fig.6D) although simultaneous down-regulation of Th2 mLN responses were not observable in recipient mice (data not shown). Lower numbers of transferred cells (1×10^6^) did not significantly alter acute-stage enteric granuloma formation (data not shown). Thus, CD4^+^CD25^+^T_regs_ cells, which expand within the mLN of chronically-infected mice, exert a suppressive effect on the development of acute-phase Th2 inflammation.

## Discussion

Our data provides both *in vitro* and *in vivo* evidence that intestinal-associated CD4^+^CD25^+^FoxP3^+^T_regs_ expand during chronic, schistosome-induced colitic inflammation. They mediate significant levels of antigen-specific Th2 suppression *in vivo* including reduced IL-4 production, eosinophil recruitment, collagen production, and an overall reduction in the size of egg-induced granulomas in the large intestine. Thus, our data demonstrates experimentally, that CD4^+^CD25^+^ T_regs_ are capable of modulating Th2 inflammation and fibrosis associated with intestinal disorders.

Whilst an expansion of gut-associated CD4^+^FoxP3^+^T_regs_ was observed following schistosome infection, this could be a product of proliferating, naturally occurring (n)T_regs_ in response to auto-antigens (*e.g.* arising from disrupted intestinal barrier), or comprise an induced population of FoxP3^+^T_regs_ recognising schistosome antigens. However, in a syngenic adoptive transfer model, numbers of nT_regs_ did not expand relative to CD4^+^ effector cells within the mLN [24], suggesting that nT_reg_ expansion does not fully account for the heightened ratio of FoxP3^+^CD4^+^ : CD4^+^ cells during chronic infection. The development of schistosome infection-induced T_regs_ is likely to be favoured by the constitutive production of intestinal signals such as TGFβ and retinoic acid [29], [30]. Intriguingly, SEA induces TGFβ1 secretion [31] and is critical for the development of auto-immune suppressing FoxP3^+^ T_regs_ following in vivo SEA injection [32]. Since we observed heightened bioactive TGFβ1 from mLN cells and within the colon at the chronic phase of infection, we speculate that TGFβ1 might have a role in the induction of gut-homing CD4^+^FoxP3^+^T_regs_ from naïve precursors. Both proportions and absolute numbers of CD4^+^FoxP3^+^T_regs_ significantly increased within the mLN during acute infection, when anti-egg Th2 responses are at their peak. Although inhibition of Treg induction by Th2 differentiation programs has been reported [33], our data would suggest that Th2 differentiation is insufficient to block outgrowth of a regulatory T cell phenotype during acute intestinal schistosomiasis.

CD4^+^FoxP3^+^T_regs_ within the mLN of mice with a chronic infection expressed elevated levels of CD103 and CCR5, both of which are associated with homing to mucosal tissues during inflammation [20], [21]. However, infection status did not alter the in vitro suppressive ability on a per-cell basis of CD4^+^CD25^+^T_regs_ from the mLN, indicating that infection-expanded CD4^+^FoxP3^+^T_regs_ and those from naïve mice share a common mechanism of Th2-effector cell suppression. Therefore, it is likely that the increased regulatory activity of CD4^+^FoxP3^+^T_regs_ *in vivo* during chronic infection is a product of either increased numbers trafficking to the site of egg deposition, or greater survival/ retention in colonic granulomas. As recipients of schistosome-expanded CD4^+^CD25^+^ cells displayed selective suppression of Th2 activity at the enteric infection site versus the mLN, this may reflect a biased homing of T_regs_ in the colon that provides increased regulatory pressure on the local granulomatous response (*i.e.* collagen synthesis and eosinophil recruitment). Taken with the observation that ablation of CD4^+^CD25^+^ cells *in vivo* significantly restores the anti-egg IL-4 and proliferative mLN cell response, these data suggest that intestinal-associated, schistosome infection-expanded FoxP3^+^ T_regs_ exert layers of Th2 suppression both within gut-draining lymph nodes and within the colonic infection site.

Our data are consistent with recent findings that CD4^+^FoxP3^+^T_regs_ constitute a partial component of the modulation of granuloma development in the liver [14], [24], [34]. We show that depletion of CD4^+^CD25^+^ cells *in vivo* does not fully reverse the Th2 hypo-responsiveness, nor do chronic enteric granulomas in treated mice fully recover the florid cellularity of the acute phase. The use of transgenic mice deficient for *foxp3* [35] could help further investigation of this phenomenon. The development of other regulatory cells such as alternatively-activated macrophages [36], IL-10-secreting non-FoxP3 cells [14], and regulatory B cells [37], may explain the partial role of CD4^+^FoxP3^+^T_regs_ in our system. T cell intrinsic anergy, for instance mediated by GRAIL signalling [38], could also account for the remaining hypo-responsiveness after depletion of CD4^+^ CD25^+^ cells. However, because the micro-environment of the intestines is favourable towards the expansion of CD4^+^FoxP3^+^T_regs_, it is possible that CD4^+^FoxP3^+^T_reg_-mediated suppression of enteric granulomas is more apparent compared with their hepatic counterparts. This could explain why colonic granulomas modulate more rapidly versus hepatic granulomas after the acute stage, and why hepatic granulomas retain a greater size and cellularity than enteric granulomas during chronic disease [19].

During experimental helminth infections, CD4^+^CD25^+^T_reg_-mediated suppression of Th2 effector responses confer a permissive state by stifling effective Th2-mediated worm attrition [23], [25], [26]. In the case of gut-helminths, this potentially operates by regulating Th2 cytokine signalling on smooth muscle contractility and epithelial cell turnover [39], [40]. Paradoxically, during *S. mansoni* infections, while Th2 granulomatous responses protect and aid survival of the host [11], [41], intact Th2 responses are also essential to propagate the parasite's life cycle, as egg transmission from the gut is impaired in the absence of T cells [42], [43], [44], IL-4 [41], or IL-4Rα signalling [45]. Nevertheless, transfer of CD4^+^CD25^+^ cells to acute stage recipients does not impinge on egg transmission in spite of a modulatory effect on IL-4 in the colon. In fact, during the chronic stage of infection, where Th2 inflammation in the colon becomes modulated, egg excretion rates are unaffected (our unpublished observations). How intestinal schistosome parasites mediate sustained, chronic egg transmission in the face of marked Th2 hypo-responsiveness remains to be identified.

Enteric helminth infections, or products derived from helminths, are gaining prominence as potential therapies to reverse the effects of inflammatory bowel disease [46]. Some ameliorating capacities may be attributed to antagonism of Th1 processes by induction of IL-4-secreting cells within intestinal tissues rather than by induction of T_regs_. Indeed, most experimental studies demonstrating the modulatory action of helminth infection used trinitrobenzene sulphate as a haptenizing agent to drive Th1 colitis reminiscent of Crohn's Disease [46]. Initiation of Th2/NKT colitis by oxazolone in conjunction with Th2-promoting helminth infection exacerbated pathology [47], further supporting that the mechanism of helminth suppression of colitis is based on Th2/Th1 antagonism. Thus, from a clinical perspective, helminth-based therapies might be considered inappropriate for ulcerative colitis or other intestinal disorders with Th2 aetiology. However, our data demonstrates that helminth infection-expanded FoxP3^+^ T_regs_ clearly regulate coincident pro-fibrotic Th2 processes in the colon. Indeed, distinct molecules released by schistosome eggs deliver triggers that polarize naïve CD4^+^ T cells towards Th2, or a regulatory phenotype [8], [9], [10], [48]. Potentially, the release of somatic molecules with regulatory potential from degrading eggs that fail to breach colonic tissues could favour T_reg_ expansion during chronic disease in the context of a TGFβ-enriched microenvironment. Exploitation of such regulatory molecules may be of benefit in the treatment of intestinal schistosomiasis or other Th2-based intestinal disorders via the expansion of T_regs_ with bystander potential.

## Supporting Information

Video S1 **Acute colonic granuloma shown in** **Fig 1F** **.**
(MOV)

Video S2 **Chronic colonic granuloma shown in** **Fig 1F** **.**
(MOV)

Video S3 **Control recipient, colonic granuloma shown in** **Fig. 6B** **.**
(MOV)

Video S4 **CD4^**+**^CD25^**+**^ recipient, colonic granuloma shown in** **Fig. 6B** **.**
(MOV)

Table S1 **Acute-stage schistosome egg tissue burdens and excretion following adoptive transfer of chronic infection-expanded CD4^**+**^CD25^**+**^ cells.**
(DOC)
