# Supplementary material for: CD4+CD25+ Regulatory Cells Contribute to the Regulation of Colonic Th2 Granulomatous Pathology Caused by Schistosome Infection
Source: PLoS Negl Trop Dis. 2011 Aug 9;5(8):e1269. doi: 10.1371/journal.pntd.0001269 (PMC3153428; doi:10.1371/journal.pntd.0001269)
Supplement: Table S1 — Acute-stage schistosome egg tissue burdens and excretion following adoptive transfer of chronic infection-expanded CD4+CD25+ cells. (DOC) [file pntd.0001269.s005.doc]

**Supplementary Data**

**Supplementary Table 1**

**Acute-stage schistosome egg tissue burdens and excretion following adoptive transfer of chronic infection-expanded CD4^+^CD25^+^ cells**

|  | Colonic eggs (x10^3^/g) | Liver eggs (x10^3^/g) | Excreted eggs  (x10^3^/g) | | |
| --- | --- | --- | --- | --- | --- |
| Time post-transfer | +28d | +28d | +14d | +21d | +28d |
| RPMI control | 0.47  (0.27-0.79) | 3.40  (2.07-5.76) | 0.25 (0.07-0.40) | 0.70  (0.54-0.95) | 0.53  (0.44-0.69) |
| 2.5x10^6^ CD4^+^CD25^+^ cells | 1.53  (0.36-2.70) | 3.49  (1.73-4.75) | 0.40  (0.24 – 0.48) | 0.88  (0.67-1.21) | 0.75  (0.36-1.18) |
